# A preliminary clinical trial to evaluate ^64^Cu-NOTA-Trastuzumab as a positron emission tomography imaging agent in patients with breast cancer

**DOI:** 10.1186/s13550-021-00746-1

**Published:** 2021-01-21

**Authors:** Inki Lee, Ilhan Lim, Byung Hyun Byun, Byung Il Kim, Chang Woon Choi, Sang-Keun Woo, Kwang Il Kim, Kyo Chul Lee, Joo Hyun Kang, Min-Ki Seong, Hyun-Ah Kim, Woo Chul Noh, Sang Moo Lim

**Affiliations:** 1 Department of Nuclear Medicine, Korea Cancer Centre Hospital, Korea Institute of Radiological and Medical Sciences, 75, Nowon-ro, Nowon-gu, Seoul, Korea; 2 Division of Applied RI, Research Institute of Radiological and Medical Sciences, Korea Institutes of Radiological and Medical Sciences, Seoul, Korea; 3 Department of Surgery, Korea Cancer Centre Hospital, Korea Institutes of Radiological and Medical Sciences, 75, Nowon-ro, Nowon-gu, Seoul, Korea

**Keywords:** HER-2, ^64^Cu-NOTA-Trastuzumab, Breast cancer, Positron emission tomography, Computed tomography

## Abstract

**Background:**

The purpose of this study was to evaluate both the biodistribution and safety of ^64^Cu-1,4,7-triazacyclononane-1,4,7-triacetic acid (NOTA)-Trastuzumab, a novel ^64^Cu-labeled positron emission tomography (PET) tracer for human epidermal growth factor receptor 2 (HER2) in patients with breast cancer.

**Methods:**

PET images at 1, 24, and 48 h after 296 MBq of ^64^Cu-NOTA-Trastuzumab injection were obtained from seven patients with breast cancer. Both the primary tumors’ and metastatic lesions’ maximum standardized uptake value (SUV_max_) was evaluated. The mean SUV_max_ (SUV_mean_) was evaluated in the other organs, including the blood pool, liver, kidney, muscle, spleen, bladder, and the lungs, as well as the bones. Moreover, the internal radiation dosimetry was calculated using the OLINDA/EXM software. Safety was assessed based on feedback regarding adverse reactions and safety-related issues within 1 month after ^64^Cu-NOTA-Trastuzumab administration.

**Results:**

^64^Cu-NOTA-Trastuzumab PET images showed that the overall SUV_mean_ values in each organ negatively correlated with time. The liver’s average SUV_mean_ values were measured at 5.3 ± 0.7, 4.8 ± 0.6, and 4.4 ± 0.5 on 1 h, 24 h, and 48 h after injection, respectively. The average SUV_mean_ blood values were measured at 13.1 ± 0.9, 9.1 ± 1.2, and 7.1 ± 1.9 on 1 h, 24 h, and 48 h after injection, respectively. The SUV_max_ of HER2-positive tumors was relatively higher than HER2-negative tumors (8.6 ± 5.1 and 5.2 ± 2.8 on 48 h after injection, respectively). Tumor-to-background ratios were higher in the HER2-positive tumors than in the HER2-negative tumors. No adverse events related to ^64^Cu-NOTA-Trastuzumab were reported. The calculated effective dose with a 296 MBq injection of ^64^Cu-NOTA-Trastuzumab was 2.96 mSv. The highest absorbed dose was observed in the liver (0.076 mGy/MBq), followed by the spleen (0.063 mGy/MBq), kidney (0.044 mGy/MBq), and heart wall (0.044 mGy/MBq).

**Conclusions:**

^64^Cu-NOTA-Trastuzumab showed a specific uptake at the HER2-expressing tumors, thus making it a feasible and safe monitoring tool of HER2 tumor status in patients with breast cancer.

***Trial registration*:**

CRIS, KCT0002790. Registered 02 February 2018, https://cris.nih.go.kr

## Background

The specific receptors that are expressed in cancer cells have been considered as targets for the treatment of tumors, resulting in an improved therapeutic performance [1]. Among them, the human epidermal growth factor receptor (HER)—which is involved in the growth of cancer cells—is a target of a representative molecular therapeutic agent [1, 2]. The overexpression of HER—an intrinsic protein tyrosine kinase—is closely related to rapid-progress tumors [3]. A member of the HER receptor family, HER2/neu (HER2), is overexpressed in breast, ovarian, bladder, prostate, and non-small cell lung cancer [3]. Recently, several therapeutic agents targeting HER2 have been developed to improve treatment outcomes, which include Trastuzumab, Lapatinib, and Pertuzumab [4].

The expression of HER2 is evaluated by tumor tissue acquisition, which entails an inevitably invasive procedure [2, 5, 6]. The discordance rate of HER2 expression between primary tumors and distant metastatic lesions is 4.9–17.7% [7]; therefore, it is necessary to re-evaluate HER2 expression in metastatic tumors. Moreover, HER2 expression may change over time after cancer develops [8], thus necessitating continuous HER2 evaluation. However, repeated biopsies are discomforting for the patient. Overcoming this limitation requires a noninvasive evaluation of HER2 expression of using radioisotopes [2, 5, 6, 9].

Various attempts have been made to noninvasively evaluate the expression of HER2 using radioisotopes, including the evaluation of the HER2 expression using single-photon emission computerized tomography (SPECT) with ^111^In-Trastuzumab [10, 11]. We investigated the possibility of evaluating HER2 expression; however, it presented both low sensitivity and limited spatial resolution [10]. To overcome these limitations, a diagnostic method using positron emission tomography (PET) has been studied. Clinical trials of antibodies such as Trastuzumab labeled with radioisotopes, including ^124^I and ^89^Zr, have been conducted [9, 11, 12], demonstrating HER2 expression capability of lesions in patients with HER2 expressing tumors [9, 11, 12]. Moreover, the use of HER2-targeted PET imaging, using ^64^Cu-tetra-azacyclododecanetetra-acetic acid (DOTA)-Trastuzumab, has been attempted [5, 6, 11, 13, 14]; related clinical trials in the USA and Japan showed effective identification of HER2 expression in breast cancer patients [5, 6, 13, 14].

We have previously developed the ^64^Cu-1,4,7-triazacyclononane-1,4,7-triacetic acid (NOTA)-Trastuzumab, targeting HER2-expressing tumors and conducted both in vitro and in vivo experiments, showing that ^64^Cu-NOTA-Trastuzumab can be used as a PET-diagnostic application for HER2-positive breast cancer [2]. Here, we evaluated the safety and pharmacokinetics of ^64^Cu-NOTA-Trastuzumab in breast cancer patients.

## Methods

### Participants

We recruited a total of seven patients with breast cancer between September 2017 and September 2019 with the following selection criteria: (1) aged 40–80 years, (2) at least one measurable lesion, (3) a histopathologically diagnosed breast cancer with a HER2 expression, and (4) an Eastern Cooperative Oncology Group score of 2 or lower.

This study was approved by the Korean Ministry of Food and Drug Safety (MFDS), and the Institutional Review Board of KIRAMS (IRB No.: KIRAMS 2017-09-006-020). All procedures were performed following the 1964 Declaration of Helsinki and its later amendments or comparable ethical standards. Informed consent was obtained from all participants. This preliminary clinical trial is registered with the Clinical Research Information Service (https://cris.nih.go.kr), registration number KCT0002790.

### Preparation of ^64^Cu-NOTA-Trastuzumab

^64^Cu-NOTA-Trastuzumab was produced from the immunoconjugate NOTA-Trastuzumab, radiolabeled with ^64^Cu from 50-meV cyclotron irradiation [2]. Briefly, Trastuzumab (Herceptin®; F. Hoffmann-La Roche, Basel, Switzerland) was dissolved in 0.1 M 4-(2-hydroxyethyl)-1-piperazineethanesulfonic acid (HEPES) buffer (pH 8.5) at a concentration of 10 mg/ml and mixed with a 20-fold molar excess of *p*–SCN-Bn-NOTA in 100% ethanol. The immunoconjugate (NOTA-Trastuzumab) was purified after an overnight incubation at 4 °C and was concentrated to 5 mg/mL with 0.1 M ammonium acetate buffer (pH 6). For radiolabeling, 370 MBq of ^64^CuCl_2_ was added to 5 mg of NOTA-Trastuzumab. The reaction mixtures were incubated at room temperature for 1 h, with a radiolabeling efficiency of > 95%. The reaction mixtures formulated with saline were sterilized by filtration through a 0.22 μm Millex GV filter (Merck Millipore, Billerica, MA, USA).

### PET protocol

The ^64^Cu-NOTA-Trastuzumab PET images were acquired using a GE Discovery 710 PET/ computed tomography (CT) (GE Healthcare, Milwaukee, WI, USA). After a 45 mg Trastuzumab intravenous injection for at least 15 min, participants were intravenously injected with ^64^Cu-NOTA-Trastuzumab (296 MBq). The mean administered activity was 278.4 ± 13.0 MBq (range 259.0–297.0 MBq). No adverse or clinically detectable pharmacologic effects were found in any of the seven participants; moreover, no significant changes either in vital signs or the results of laboratory studies were reported. PET images were obtained at 60 min after intravenous injection of ^64^Cu-NOTA-Trastuzumab. Delayed PET images were obtained between 20 and 25 h, and 46 and 49 h after ^64^Cu-NOTA-Trastuzumab injection. All participants were scanned from the mid-thigh to the vertex of the skull.

^18^F-Fluorodeoxyglucose (FDG) PET/CT was performed 1 day before the ^64^Cu-NOTA-Trastuzumab PET. After 6 h of fasting, 370 MBq of ^18^F-FDG was intravenously injected. The blood glucose level before ^18^F-FDG injection did not exceed 7.2 mmol/L. One h after injection, PET images were acquired using GE Discovery 710 PET/CT (GE Healthcare, Milwaukee, WI, USA).

PET images were reconstructed using a conventional iterative algorithm and ordered-subsets expectation–maximization with parameters of four iterations and eight subsets. For attenuation correction, CT scans were obtained (130 kVp, 30 mA, 0.6 s/CT rotation, and 6 pitch), after voiding the bladder.

### Radiation dosimetry

The internal dosimetry of ^64^Cu-NOTA-Trastuzumab was evaluated using accumulated radioactivity in PET images. The organ time-activity curve of radioactivity in the target region (ID) divided by target mass (g) was acquired from each organ to calculate residence time. The time-activity curve was expressed by three time points at 1, 24, and 48 h. The residence times were calculated by accumulated radioactivity divided by subject administered activity. The S value of the source-to-target region energy, deposited per unit mass, was calculated using OLINDA/EXM version 1.1 software, using an adult female as the model. The organ-absorbed doses were calculated as the self-dose and cross-dose from each organ region.

### Biodistribution

The biodistribution of ^64^Cu-NOTA-Trastuzumab was evaluated using the maximum standardized uptake value (SUV_max_) and the mean standardized uptake value (SUV_mean_) from the three sequential PET images using GE AW software (GE Healthcare, Milwaukee, WI, USA). Regarding normal-organ distribution, the blood, liver, kidney, muscle, spleen, bladder, lung, and bone were analyzed. Regarding tumors, the primary tumor, metastatic lymph nodes (LNs), and metastatic bone lesions were also evaluated. A 2–3 cm ellipsoidal volume of interest was drawn inside the organ on the PET images to calculate the SUV.

The lesion-to-background ratios were calculated to test the degree of ^64^Cu-NOTA-Trastuzumab uptake at the lesion sites. The SUV_mean_ either of the liver or blood was used as the background. The SUV_max_ of the breast tumors, metastatic LNs, and metastatic bone lesions was used for lesion assessment.

### Safety

Safety was assessed both before and after ^64^Cu-NOTA-Trastuzumab administration, acquiring feedback—including adverse reactions and other safety-related issues—1 month later. Adverse events, vital signs, physical examination data, and laboratory test results were all considered in the safety evaluation.

## Results

### Participant characteristics

Seven patients with breast cancer were recruited. One screened participant was excluded, because of a failure to produce radioisotopes, thus evaluating a total of six participants.

During the initial diagnosis, the immunohistochemistry (IHC) results from core needle biopsy found three patients with HER2-positive tumors and another three with HER2-negative tumors; however, after neo-adjuvant chemotherapy, the final IHC results from tumor excision showed two patients with HER2-positive tumors and four with HER2-negative tumors. One patient with an IHC score of 3+ from core needle biopsy had the result changed into IHC score 1+ from excision after neoadjuvant chemotherapy, thus classifying this patient as HER2-negative.

Patient cancer staging was checked from IIA to IV. The period between histology evaluation and ^64^Cu-NOTA-Trastuzumab imaging was 1–3 months. All participants—except for participant 1—underwent neoadjuvant chemotherapy with adriamycin and cyclophosphamide before the ^64^Cu-NOTA-Trastuzumab PET/CT scan. The tumor size at the time of ^64^Cu-NOTA-Trastuzumab PET/CT scan was measured at 1.7–4.0 cm.

Detailed participant characteristics are described in Table 1.

**Table 1:** Participant characteristics

Subject no	Age (years)	Histology	Stage	IHC score^*^ (CNB)	SISH	IHC score^†^ (excision)	Neoadjuvant chemotherapy	History of Trastuzumab treatment	Interval from CNB to ^64^Cu-NOTA-Trastuzumab (months)	Tumor size (cm)
1	45	IDC	IV	3+	N/A	N/A	–	–	1	7.3
2	42	IDC	IIIB	3+	N/A	3+	AC #3	–	2	2.4
3	46	IDC	IIIA	3+	N/A	1+	AC #4	–	3	1.7
4	45	IDC	IIA	–	N/A	–	AC #4	–	3	2.2
5	56	IDC	IIIB	2+	–	1+	AC #4	–	3	14.0
6	54	IDC	IIB	–	N/A	–	AC #3	–	2	4.6

*IHC* immunohistochemistry, *SISH* silver-enhanced in situ hybridization, *CNB* core needle biopsy, *IDC* invasive ductal carcinoma, *N/A* not applicable, *AC* anthracycline, and cyclophosphamide

^*^IHC score with core needle biopsy at the initial diagnosis

^†^IHC score with tumor excision after neoadjuvant chemotherapy

### Safety

No adverse events related to the use of ^64^Cu-NOTA-Trastuzumab were observed.

### Radiation dosimetry

**Fig. 1:**
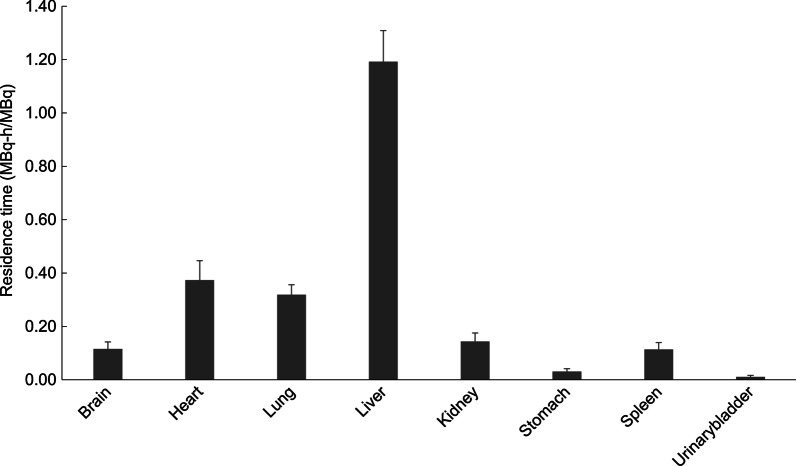
Residence time derived from serial positron emission tomography images (PET). Mean organ residence times (± standard deviation) for ^64^Cu-NOTA-Trastuzumab

The estimated radiation-absorbed dose for each organ is described in Table 2. The organ with the highest absorbed doses was the liver, with 0.076 ± 0.007 mGy/MBq. The effective dose was calculated as 0.010 ± 0.001 mSv/MBq. Therefore, when injected with 296 MBq of ^64^Cu-NOTA-Trastuzumab, the effective dose was 2.96 mSv. Figure 1 shows the residence time for each organ.

**Table 2:** Dosimetry of ^64^Cu-NOTA-Trastuzumab (OLINDA)

Organ	Absorbed dose (mGv/MBq)		
	^64^Cu-NOTA-Trastuzumab	^64^Cu-DOTA-Trastuzumab [6]	[^89^Zr]Trastuzumab [9]
Adrenals	0.005 ± 0.001	0.031 ± 0.004	0.80
Brain	0.009 ± 0.002	0.015 ± 0.003	0.39
Breasts	0.002 ± 0.000	0.020 ± 0.001	0.42
Gallbladder wall	0.006 ± 0.001	0.035 ± 0.008	0.86
LLI wall	0.000 ± 0.000	0.018 ± 0.002	0.58
Small intestine	0.001 ± 0.000	0.019 ± 0.001	0.57
Stomach wall	0.008 ± 0.002	0.024 ± 0.002	0.63
ULI wall	0.002 ± 0.000	0.022 ± 0.002	0.65
Heart wall	0.042 ± 0.008	0.340 ± 0.046	1.11
Kidneys	0.044 ± 0.009	0.103 ± 0.034	1.23
Liver	0.076 ± 0.007	0.237 ± 0.117	1.63
Lungs	0.034 ± 0.004	0.057 ± 0.070	0.59
Muscle	0.001 ± 0.000	0.023 ± 0.006	0.49
Ovaries	0.001 ± 0.000	0.018 ± 0.002	0.59
Pancreas	0.005 ± 0.001	0.032 ± 0.003	0.78
Red marrow	0.001 ± 0.000	0.017 ± 0.001	0.69
Osteogenic cells	0.001 ± 0.000	0.035 ± 0.001	0.79
Skin	0.001 ± 0.000	0.015 ± 0.001	0.34
Spleen	0.063 ± 0.010	0.142 ± 0.040	0.86
Thymus	0.003 ± 0.000	0.030 ± 0.002	0.57
Thyroid	0.000 ± 0.000	0.016 ± 0.001	0.43
Urinary	0.003 ± 0.001	0.023 ± 0.006	0.42
Uterus	0.001 ± 0.000	0.018 ± 0.002	0.58
Total body	0.004 ± 0.000	0.029 ± 0.004	0.55
Effective dose (mSv/MBq)	0.010 ± 0.001	0.036 ± 0.009	0.61

### Normal-organ biodistribution and tumor uptake

**Fig. 2:**
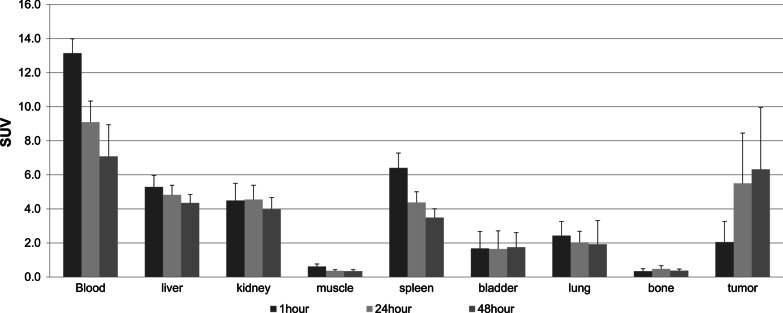
Mean standardized uptake value (SUV_mean_) with a standard error of ^64^Cu-NOTA-Trastuzumab in normal organs and maximum standardized uptake value (SUV_max_) with a standard error of ^64^Cu-NOTA-Trastuzumab in tumors

**Fig. 3:**
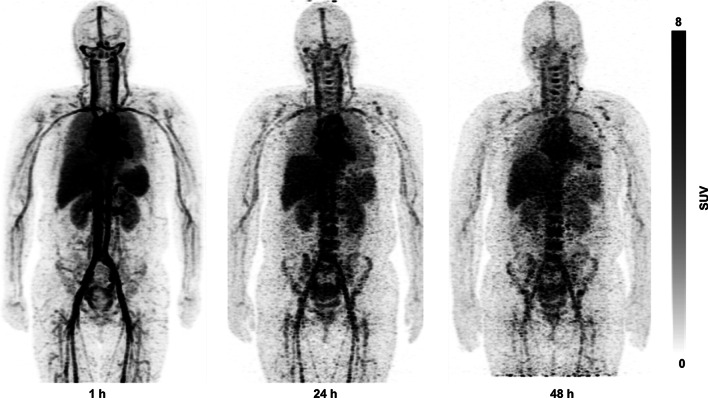
Maximum intensity projection images of ^64^Cu-NOTA-Trastuzumab PET at 1, 24, and 48 h after injection

The uptakes of ^64^Cu-NOTA-Trastuzumab in normal organs, including blood pool, liver, kidneys, muscles, spleen, bladder, lungs, and bones, are presented in Fig. 2 as SUV_mean_. Maximum intensity projection (MIP) images of participant 1 show the whole-body distribution of ^64^Cu-NOTA-Trastuzumab in Fig. 3.

The uptake of ^64^Cu-NOTA-Trastuzumab in the blood showed a high value at 1 h after injection and a decreasing pattern over time. The SUV_mean_ of the liver also showed a high value at 1 h after injection and a gradual decrease over time. The bladder’s SUV_mean_ was maintained at a low value at 1–48 h after injection. Overall, the uptakes of ^64^Cu-NOTA-Trastuzumab in the blood, liver, kidneys, and spleen were relatively high.

**Fig. 4:**
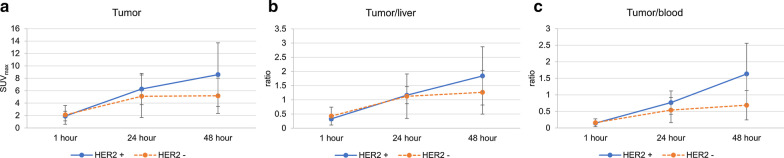
The changes of SUV_max_ (**a**), tumor-to-liver ratio (**b**), and tumor-to-blood pool ratio (**c**) of HER2-positive and HER2-negative tumors over time

The average values of SUV_max_ for HER2 positive tumors were: 1.9 ± 0.8 at 1 h; 6.3 ± 2.5 at 24 h; and 8.6 ± 5.1 at 48 h after ^64^Cu-NOTA-Trastuzumab injection. In the case of HER2 negative tumors, the average values of SUV_max_ were: 2.1 ± 1.5 at 1 h; 5.1 ± 3.4 at 24 h; and 5.2 ± 2.8 at 48 h after ^64^Cu-NOTA-Trastuzumab injection. The lesion-to-liver ratios at 48 h after injection were 1.8 ± 1.0 and 1.3 ± 0.8 for HER2 positive and negative tumors, respectively. The lesion-to-blood ratios at 48 h were 1.6 ± 0.9 and 0.7 ± 0.4 for HER2 positive and negative tumors, respectively. Figure 4 shows changes in both the SUV_max_ of the tumors and tumor-to-background ratios depending on time. Overall, the values of both SUV_max_ and tumor-to-background ratios were higher in HER2-positive tumors than HER2-negative tumors. The values of HER2-positive tumors increased over time up to 48 h after injection; however, HER2-negative tumors did not show the same increase in uptake values observed for the HER2-positive tumors.

**Fig. 5:**
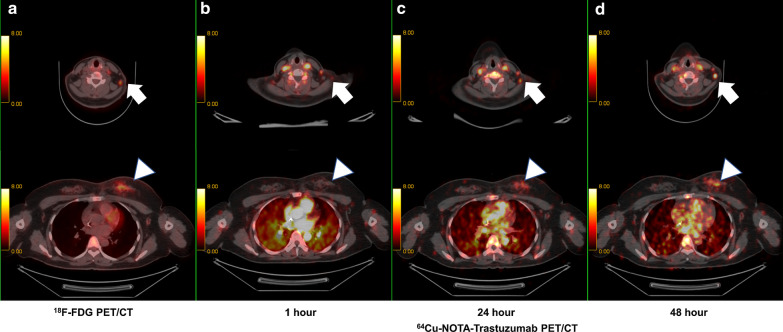
^64^Cu-NOTA-Trastuzumab PET images of HER2-positive breast cancer (arrowheads, lower row) and metastatic lymph node (arrows, upper row). The primary tumor and metastatic lymph nodes were clearly observed by ^18^F-FDG PET/CT (**a**) and ^64^Cu-NOTA-Trastuzumab PET/CT (**b**–**d**). In HER2-expressing lesions, the uptakes of NOTA increased over time to 48 h after injection

Participant 1’s images of ^64^Cu-NOTA-Trastuzumab PET/CT are shown in Fig. 5; Fig. 5a (first column) is the ^18^F-FDG PET/CT image and Fig. 5b–d (second to fourth columns) shows ^64^Cu-NOTA-Trastuzumab PET/CT images regarding time (on 1, 24, and 48 h, respectively). Participant 1 was a 45-year-old, left breast cancer patient with multiple metastatic lymph nodes and bones. The tumor showed a HER2-positive expression (HER2 3 +). The upper-row arrows show the metastatic lymph node on the left side of the neck. High ^18^F-FDG uptake was shown in the ^18^F-FDG PET/CT (Fig. 5a upper row), and the uptakes of ^64^Cu-NOTA-Trastuzumab increased over time in the same lesion with the FDG PET/CT images (Fig. 5b–d upper row). The SUV_max_ of the metastatic lymph node was: 1.2 at 1 h; 6.5 at 24 h; and 11.6 at 48 h after injection. The lower row of Fig. 5 shows the primary tumor in the left breast. ^18^F-FDG uptake appears in the left breast cancer (Fig. 5a, lower row, arrowhead). The uptakes of ^64^Cu-NOTA-Trastuzumab were also found in the same lesion with increases over time (Fig. 5b–d, lower row, arrowhead). The SUV_max_ of the primary tumor was: 2.2 at 1 h; 5.8 at 24 h; 9.7 at 48 h after injection.

## Discussion

This study demonstrated that a novel HER2-targeted PET ligand, ^64^Cu-NOTA-Trastuzumab, was safe, had no adverse effects, and provided a relatively low exposure to radiation (2.96 mSv from a 296-MBq injection). Moreover, the uptakes of ^64^Cu-NOTA-Trastuzumab were observed in the HER2-expressing tumors including primary breast cancer, metastatic lymph nodes, and metastatic bones.

Due to the tumor heterogeneity, HER2 expression could differ between the primary and the metastatic lesions and may vary depending on disease progression [7, 8]. Therefore, evaluating the HER2 expression before HER2-targeted therapy to enhance the patients’ treatment efficacy is vital.

^64^Cu-DOTA-Trastuzumab PET—an evaluation method of HER2 expression in a noninvasive procedure—was a feasible modality based on clinical trials [5, 6, 13, 14]. Compared to other PET agents—such as ^89^Zr and ^124^I for evaluating HER2 expression—^64^Cu has the benefit of reducing radiation exposure with a relatively short half-life [2]. Moreover, PET/CT can be performed in an outpatient setting, thus making it advantageous. Previous studies have reported that ^64^Cu-DOTA-Trastuzumab PET has difficulty in distinguishing either metastatic lesions or tumors, in the liver and around the blood vessels due to the high physiologic uptakes of ^64^Cu-DOTA-Trastuzumab [6], which are found in both the liver and blood [15]. However, in a chelator comparison study for ^64^Cu labeled compound administration in vivo, a reduction in non-specific uptake was observed for the ^64^Cu-NOTA compound compared to the ^64^Cu-DOTA compound [16]. Further, the ^64^Cu-NOTA compound showed considerably lower accumulation in the liver than the ^64^Cu-DOTA compound [17]. Therefore, we developed ^64^Cu-NOTA-Trastuzumab to achieve improved PET imaging for HER2 expression, compared to ^64^Cu-DOTA-Trastuzumab [2].

Previous studies with ^64^Cu-DOTA-Trastuzumab [6, 18] evaluated breast cancer patients with only HER2-positive tumors; however, despite the limitation of the small sample number, our study included both patients with HER2-positive and negative tumors. In our study, HER2-positive tumors showed a high ^64^Cu-NOTA-Trastuzumab uptake, whereas HER2-negative tumors did not. Adding to the previous preclinical results [2], this study suggested that ^64^Cu-NOTA-Trastuzumab can effectively differentiate HER2-positive from HER2-negative tumors in patients with breast cancer.

The biodistribution of ^64^Cu-NOTA-Trastuzumab in normal organs showed high uptakes in both the blood and liver, in line with the in vivo study [2]. Nevertheless, ^64^Cu-NOTA-Trastuzumab showed that it is advantageous regarding the relatively low uptake in the liver compared to ^64^Cu-DOTA-Trastuzumab. Conversely, blood uptake is relatively higher than ^64^Cu-DOTA-Trastuzumab. The relatively low uptake in the liver could be due to the stable copper-binding ability of NOTA, potentially reducing free copper accumulation; however, a direct comparison of the results is limited, as the liver uptake between ^64^Cu-NOTA-Trastuzumab and ^64^Cu-DOTA-Trastuzumab was compared in different tumor models [19]. ^64^Cu-NOTA-Trastuzumab showed a relatively low effective dose (0.014 mSv/MBq) compared to the latest radiolabeled Trastuzumab studies (^64^Cu-DOTA-Trastuzumab, 0.036 mSv/MBq; ^89^Zr-Trastuzumab, 0.61 mSv/MBq), potentially reducing patient radiation exposure [6, 9].

The uptake of ^64^Cu-NOTA-Trastuzumab in HER2-expressing tumors was not observed 1 h after injection. Nevertheless, the specific uptake increased 24 h after injection, and a further increase of specific uptake of ^64^Cu- NOTA-Trastuzumab in HER2-expressing tumors could be observed after 48 h following the injection, showing a distinctive feature from the background. It is suggested that ^64^Cu- NOTA-Trastuzumab PET at 48 h after injection can evaluate the HER2 expression in the clinical setting. Mild diffuse uptakes were also observed in the tumors with a negative expression of HER2, as HER2 expression is not all-or-none as determined by the immunohistochemistry methods [5, 20]. Namely, even if the tumor’s HER2 expression is negative, its SUV_max_ positively correlates to the tumor IHC score [5, 20]. Therefore, it is important to carefully set the cut-off values of SUV_max_ or SUV ratio between the tumor and the background to determine HER2 expression using a ^64^Cu-NOTA-trastuzumab PET image.

This study has some limitations. First, the number of enrolled participants was relatively small. Second, since all the patients—except for participant 1—underwent neoadjuvant chemotherapy, the SUV of tumors might have reflected tumor cell suppression. Therefore, the SUV might be lower than expected. Namely, regarding participants 2 and 3, with HER2 positive tumors as an initial diagnosis, the ^18^FDG PET/CT—performed before and after neoadjuvant chemotherapy—showed both decreased tumor size and metabolic activity after chemotherapy (data not shown). Therefore, the SUV of HER2 positive tumors on ^64^Cu-NOTA-Trastuzumab PET could have been underestimated. Consequently, a further study with a larger sample size and ^64^Cu-NOTA-Trastuzumab PET before neoadjuvant chemotherapy is needed to evaluate the exact efficacy of ^64^Cu-NOTA-Trastuzumab PET imaging.

## Conclusion

This preliminary clinical trial showed that ^64^Cu-NOTA-Trastuzumab PET is both safe and feasible. ^64^Cu-NOTA-Trastuzumab showed a specific uptake at the HER2-expressing tumors with a relatively low liver uptake. ^64^Cu-NOTA-Trastuzumab can be used to evaluate radiation dosimetry and prediction of treatment-response in targeted therapy for HER2-positive breast cancer with HER2-targeted therapy.

## Data Availability

The datasets used and/or analyzed during the current study are available from the corresponding author on reasonable request.

## Abbreviations

^64^Cu-DOTA-Trastuzumab: ^64^Cu-tetra-azacyclododecanetetra-acetic acid-Trastuzumab
^64^Cu-NOTA-Trastuzumab: ^64^Cu-1,4,7-triazacyclononane-1,4,7-triacetic acid-Trastuzumab PET: Positron emission tomography
CT: Computed tomography
FDG: ^1^^8^F-Fluorodeoxyglucose
HER2: Human epidermal growth factor receptor 2
SPECT: Single-photon emission computerized tomography
SUV_max_: Maximum standardized uptake value
SUV_mean_: Mean SUV_max_
